# Molecular containment of iron source inhibits larval survival of *Schistosoma mansoni* and egg-laying behavior of the female adult worms via ovarian atrophy

**DOI:** 10.1186/s41182-025-00800-x

**Published:** 2025-09-02

**Authors:** Takashi Kumagai, Rieko Shimogawara, Akira Wada

**Affiliations:** 1 Department of Health Sciences, Nippon Bunri University, 1727 Ichiki, Oita-shi, Oita, 870-0397 Japan; 2 Department of Parasitology and Tropical Medicine, Institute of Science Tokyo, 1-5-45 Yushima, Bunkyou-ku, Tokyo, 113-8510 Japan; 3 RIKEN Center for Integrative Medical Sciences, 1-7-22 Suehiro-cho, Tsurumi-ku, Yokohama, Kanagawa 230-0045 Japan

**Keywords:** *Schistosoma mansoni*, Antischistosomal drug, Iron source, Phenanthroline-based compound

## Abstract

**Background:**

Schistosomiasis is a neglected tropical disease caused by parasitic flatworms of the genus *Schistosoma*. Currently, praziquantel is the only medication available for treating schistosomiasis. However, crucial issues regarding drug resistance, reinfection, and prevention remain unresolved. Therefore, it is indispensable to develop new antischistosomal drugs, whose mechanisms of action are distinct from that of praziquantel. This diversification in treatment is vital to promote the eradication of schistosomiasis.

**Methods:**

In this study, to explore the untapped antischistosomal compounds against *Schistosoma* species, which have diverse life cycles, we initially investigated the effects of a series of phenanthroline-based compounds (PHN-*X*) with iron-binding affinity on the survival capacity of *Schistosoma mansoni* larvae and egg production by paired adult worms in vitro. Subsequently, we examined the impacts of PHN-*X* on the egg production and fecundity of female adult worms in vivo, following oral administration of them to mice infected with *S. mansoni* cercariae. Finally, we conducted a morphological analysis of the reproductive organs of the female adult worms after treating *S. mansoni*-infected mice with a newly identified compound with high anti-fecundity effect.

**Results:**

The assay for determining IC_50_ and IC_90_ values against schistosomula indicated that PHN-*X* effectively led to larval death, unlike deferoxamine and praziquantel. The larvicidal activity depended on the strength of the coordination bonds between its nitrogen atoms and an iron ion. Furthermore, PHN-*X* with electron-donating groups substantially inhibited the egg-laying behavior of paired adult worms in vitro. Notably, orally administrating PHN-(OMe)_2_ with two methoxy groups to *S. mansoni*-infected mice decreased the egg production rate of the female adult worms. The analyses of the ovarian area and the reaction of PHN-(OMe)_2_ with iron ions indicated that containment of Fe(II) ions caused abnormal ovarian atrophy, contributing to the expression of its anti-fecundity effect in vivo.

**Conclusions:**

PHN-(OMe)_2_, which has an affinity for Fe(II) ion-binding affinity, significantly affected the survival of larvae and egg-laying behavior of female adult worms. Thus, the strategy for containing the iron source required by *S. mansoni* could offer valuable insight for developing new drugs to diversify the treatment options for schistosomiasis.

**Supplementary Information:**

The online version contains supplementary material available at 10.1186/s41182-025-00800-x.

## Background

The genus *Schistosoma* is responsible for schistosomiasis as a neglected tropical disease (NTD), resulting in approximately 250 million cases worldwide [1]. In 2019, it was suspected that 128.3 million school-aged children in 51 countries were infected with *Schistosoma* species; however, only 67.2% of them received treatment for schistosomiasis [2]. Consequently, the World Health Organization (WHO) declared public health goals in the roadmap for NTDs 2021–2030, aiming to eliminate schistosomiasis as a public health problem and reduce heavy egg-patent intensity in *Schistosoma* infections to less than 1% [3]. In 2022, the WHO issued new guidelines to update the global public health strategy against schistosomiasis, offering six recommendations [4]. These guidelines included expanding the target population for preventive chemotherapy from primarily school-age children to all individuals (2 years and older). In addition, lowering the threshold for the prevalence of annual preventive chemotherapy and increasing the frequency of adequate treatment were included. Currently, the main strategy for controlling schistosomiasis is mass drug administration using the antischistosomal drug, praziquantel (PZQ) [5, 6]. However, significant challenges associated with drug resistance, reinfection, prevention, and diagnosis still need to be addressed [6–8].

The life cycle of *Schistosoma* species is quite complex [1]: Cercariae are released into water from the intermediate host, *Biomphalaria glabrata*, and invade the human body through the skin. While transforming into schistosomula, the larvae migrate from the blood vessels into the lungs. After propagation, they travel from the aortic system to the portal vein through the liver, developing into adult worms. Subsequently, male and female adult worms pair up to sexually mature and produce eggs. The uptake and ingestion of erythrocytes, which contain large amounts of hemoglobin, are crucial parts of this process to acquire the nutrients and iron source for worm growth, sexual maturation, and egg-laying [9, 10]. The eggs laid by paired adult worms embolize in the liver and intestinal wall, leading to various diseases, such as cirrhosis, portal hypertension, and sepsis [11]. Currently, PZQ is effective against the adult stage after egg production [12]. Meanwhile, artemisinin derivatives (ARTs), which are used for treating malaria caused by *Plasmodium* spp. parasites, can kill some of the larvae, but not the adult worms [13]. Therefore, while PZQ is applied for treatment in the post-diagnostic phase, ARTs could be used in the pre-patent phase [14]. However, since most of the endemic areas of schistosomiasis overlap with those of malaria, mass drug administration of ARTs is no longer recommended to prevent the emergence of drug-resistant malaria parasites.

Recent study has shown that *Schistosoma* parasites express iron transporters at the larval stage to supply iron ions and also at the adult stage to initiate egg production [15]. Moreover, administrating a natural compound with Fe(III)-binding affinity, deferoxamine (DFO), to mice infected with these parasites led to a decrease in the fecal egg excretion and an increase in the percentage of dead eggs [16]. Hence, the iron ions obtained from the digestion of hemoglobin in erythrocytes are essential for the development and ovipositional behavior of schistosomes.

**Fig. 1:**
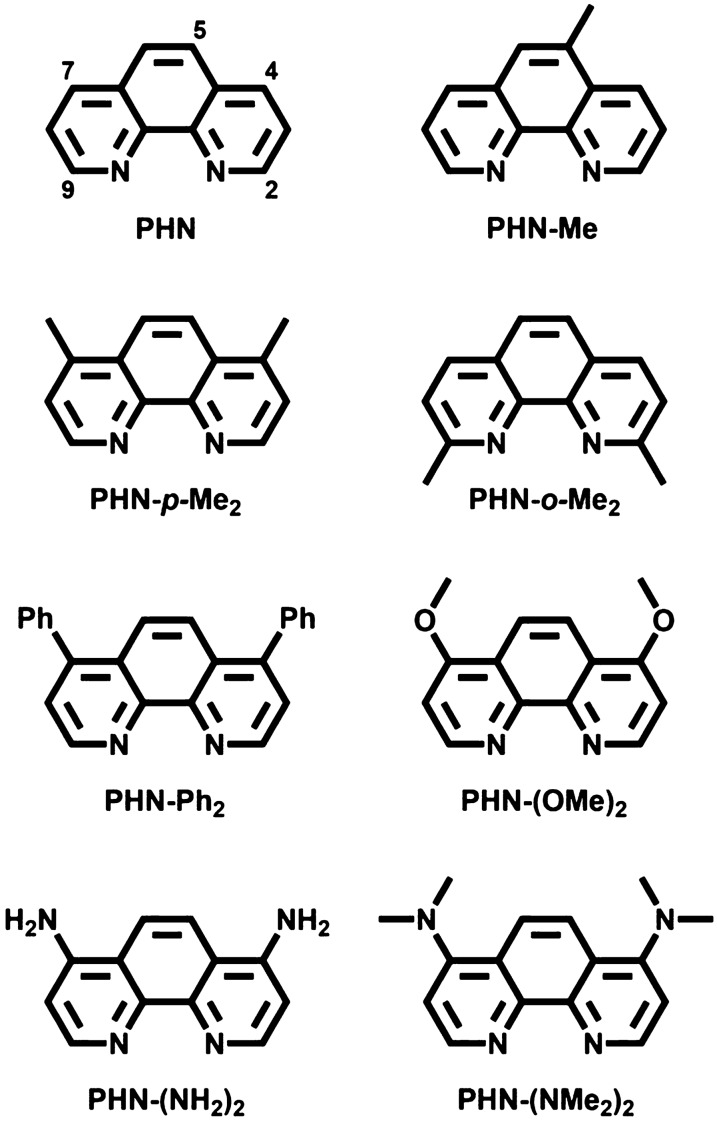
Molecular structures of phenanthroline-based compounds with various substitutional groups, PHN-*X*

We have previously reported that iron-targeting compounds possess in vitro and in vivo antiamebic activities against the infectious protozoan parasite *Entamoeba histolytica* [17]. A synthetic compound with Fe(II)-binding affinity, 1,10-phenanthroline (PHN, Fig. 1), showed a strong inhibitory effect on the growth of the parasites that acquire nutrients and iron ions by ingesting erythrocytes. In addition, when PHN was administered to hamsters infected with *E. histolytica* as animal models of amebiasis, the compound exerted a potent therapeutic efficacy, completely curing the liver abscesses without any adverse effects. These findings led us to hypothesize that synthetic compounds with a phenanthroline structure could impact the life cycle of schistosomes, which obtain iron ions by metabolizing hemoglobin in erythrocytes.

In this study, to explore the untapped antischistosomal compounds targeting diverse stages of *Schistosoma* species, we investigated the effects of a series of phenanthroline-based compounds, PHN-*X* (Fig. 1), on the survival capacity of *Schistosoma mansoni* larvae and egg production of paired adult worms in vitro. Subsequently, we assessed the impacts of PHN-*X* on the life cycle of adult worms, egg production, and fecundity of female adult worms in vivo by orally administrating these compounds to mice infected with *S. mansoni* cercariae. Furthermore, after determining the optimal dosing conditions under which PHN-(OMe)_2_ maximizes its anti-fecundity effect in *S. mansoni*-infected mice, we conducted a morphological analysis of the reproductive organs derived from female adult worms. According to the data and observations, we validated that the potential of containing iron ions, which are essential for the larvae and female adult worms, can be used for developing a new antischistosomal drug with a mode of action entirely different from those of PZQ and ARTs.

## Materials and methods

### Ethic statements

The Institutional Animal Care and Use Committee of Tokyo Medical and Dental University (TMDU) (A2021-131A) approved all animal experiments, which were conducted under the Department of Parasitology and Tropical Medicine, TMDU.

### Materials

Deferoxamine mesylate salt (Sigma-Aldrich); 1,10-Phenanthroline monohydrate (Tokyo Chemical Industry); 5-Methyl-1,10-phenanthroline (Tokyo Chemical Industry); 4,7-Dimethyl-1,10-phenanthroline (Tokyo Chemical Industry); 3,4,7,8-Tetramethyl-1,10-phenanthroline (Tokyo Chemical Industry); 2,9-Dimethyl-1,10-phenanthroline hydrochloride monohydrate (Tokyo Chemical Industry); 4,7-Diphenyl-1,10-phenanthroline (Sigma-Aldrich); 4,7-Dimethoxy-1,10-phenanthroline (Sigma-Aldrich); 1,10-Phenanthroline-4,7-diamine (Enamine); N4,N4,N7,N7-Tetramethyl-1,10-phenanthroline-4,7-diamine (Enamine); Iron(II) sulfate heptahydrate (FUJIFILM Wako Pure Chemicals). All other reagents obtained from commercial sources were used as received unless otherwise stated.

### Maintenance of *S. mansoni* using animals

According to previously described methods [18, 19], to maintain the life cycle of *S. mansoni*, *Biomphalaria glabrata* as an intermediate host (Puerto Rico strain), was individually infected with eight miracidia. The infected snails were kept in a thermostatic chamber at 28 °C for over 2 months and subsequently exposed to light to release the cercariae. Moreover, 6-week-old ICR mice (SLC, Hamamatsu, Japan) were infected by placing their tails in a tube containing 180 cercariae in tap water.

Following the Institutional Animal Care and Use Committee of TMDU (2010002C2), the infected mice were kept in a controlled temperature and humid environment with a 12:12 h light/dark cycle and had free access to food and water. Humane endpoints were considered when severe pain, suffering, excessive distress, or impending death was observed in any animal. All mice were euthanized using CO_2_ gas inhalation after the experiments. The status of the mice was monitored daily using a composite score, including vitality, fur quality, secretions, mobility, dyspnea, neurological signs, ascites, and their ability to ingest food or water. All mice were handled according to the ARRIVE guidelines and relevant regulations.

### Preparation of schistosomula and adult worms

Schistosomula were appropriately prepared as follows: over 1000 cercariae obtained from the infected snails after exposure to light, were collected in tubes through centrifugation. The cercariae were passed through a syringe attached to a 20G needle approximately 10 times to produce mechanically transformed schistosomula. The larvae were subsequently placed in 0.2 mL of RPMI1640 medium in a 96-well plate and incubated for 24 h at 37 °C in an atmosphere containing 5% CO_2_. Adult worms were collected from euthanized mice at 7-week post-infection and placed in 2 mL of RPMI1640 containing 10% FBS (Thermo Fisher Science, USA), streptomycin, penicillin, and l-glutamine (Thermo Fisher Science) in 12-well plates. After incubation for 24 h at 37 °C in an atmosphere containing 5% CO_2_, all worms were washed thrice with the conditioned medium and were then used for the experiments.

### Assessment of in vitro larvicidal activities of the compounds

The prepared schistosomula were placed in a 96-well plate (20–180 larvae/well) and the number of viable larvae was counted. Next, the compounds were added to individual wells and adjusted to final concentrations of 50, 10, 5, 1, 0.5, 0.1, and 0.05 μM. After incubation for 72 h at 37 °C in an atmosphere containing 5% CO_2_, the number of larvae was counted using previous methods [20, 21]. Dead larvae were identified based on their characteristic lack of movement and a disintegrated or completely crushed outer tegument. The IC_50_ and IC_90_ values were calculated based on the curves obtained from viability plots.

### Assessment of in vitro anti-fecundity effects of the compounds

Surviving adult worm pairs were placed in a 24-well plate (three pairs/well), and 1 mL of culture medium was added to each well [18]. Subsequently, the compounds were added at the concentration of 5 µM. To promote egg production, 1 × 10^8^ erythrocytes obtained from mice were added, and the solutions were adjusted to 2 mL by adding more culture medium. After incubation for 72 h at 37 °C in an atmosphere containing 5% CO_2_, the adult worms were removed, and 1 mL of the supernatant was discarded. Then, 2 mL of distilled water was added to each well, and the samples were incubated at room temperature for 1 h to lyse erythrocytes. After adding 1 mL of 50 mM NaOH and incubation at 4 °C overnight, the number of eggs was counted using inverted microscope to calculate the egg production rates.

### Assessment of in vivo bio-activities of the compounds

Female 6-week-old BALB/c mice were infected with 180 cercariae (five mice/group). At 2-, 4-, and 6-week post-infections, 0.1 mL of the solution containing compounds dissolved in olive oil was orally administered to each mouse for three consecutive days. After euthanizing the mice at 8-week post-infection, adult worms were collected through perfusion, and the liver and intestinal tracts were removed. The obtained male and female adult worms were separately counted to determine their survival rates. Simultaneously, the liver and intestinal tracts were completely lysed using 4% KOH solution at 37 °C overnight with shaking. After treating the solution with distilled water, 10 µL of the resulting solution was placed on a glass slide, and the eggs were counted under a microscope to calculate the egg production and fecundity rates.

### Observation of the ovaries of female adult worms

Ten female adult worms were collected from the groups of mice treated with or without PHN-(OMe)_2_ at 6-week post-infection. The adult worms were fixed for 1 week using a solution of 10% formalin, 48% alcohol, and 2% glacial acetic acid. Afterwards, the worms were stained overnight with acetocarmine solution (FUJIFILM Wako, Osaka, Japan). They were then destained in 70% acidic ethanol and were dehydrated in a graded ethanol series (70, 90, and 100%). Subsequently, the worms were cleared in a 50% xylene solution diluted in 100% ethanol for 1 min, and were mounted on slides using Bioleit (Okenshoji Co., Ltd., Japan). Finally, images were acquired using a Leica STELLARIS 5 confocal laser scanning microscopy, equipped with a 40x (NA = 1.25) oil-immersion objective and an argon laser at 488 nm as the excitation source. Images were collected as single stacks from at least ten individuals in each group. The size of the ovaries was determined by measuring the area of the images using Fiji software and calculating the means.

### Electrospray ionization mass (ESI–MS) spectroscopic analysis

The reaction of PHN-(OMe)_2_ and Fe(II) ions was initiated by mixing PHN-(OMe)_2_ (5.0 mM) and FeSO_4_ (1.67 mM) in a DMSO solution that has been degassed to remove dissolved dioxygen. The solution containing the generated iron complex was exposed to molecular dioxygen and diluted with MeOH to prepare the sample. The chemical composition of the iron complex was then determined using an ESI–MS spectrophotometer (Q Exactive, Thermo Fisher Scientific).

### Statistical analysis

Statistical comparisons were carried out using Student’s *t* test to evaluate the differences between treated and untreated groups. Differences were considered significant at *p* < 0.05 at a 95% confidence interval.

## Results and discussion

### Phenanthroline-based compounds exhibit high larvicidal activity against schistosomula of *S. mansoni*

DFO is a siderophore produced by the soil bacterium *Streptomyces pilosus*, which can tightly bind to an Fe(III) ion through the negatively charged oxygen atoms of three hydroxamic acid groups. We first investigated in vitro larvicidal activity of DFO against the schistosomula of *S. mansoni*. As shown in Table 1, the IC_50_ and IC_90_ values of DFO indicated that the natural compound did not affect the life actions of the larvae, even though it inhibited fecal egg excretion by the adult worms of schistosomes in vivo [16]. This lack of effect may be due to ineffective penetration of DFO into the larval membranes resulting from its multiple negative charges and bulky structure. In the previous study, a similar phenomenon was observed, where DFO barely inhibited the growth of *Plasmodium falciparum* at the high concentration (IC_50_ = 17.3 μM) [22]. To exclude these disadvantageous properties, we selected an electrochemically neutral compound with specific Fe(II) ions, PHN (Fig. 1), and assessed it similarly. In contrast to DFO, the IC_50_ and IC_90_ values of PHN revealed that the synthetic compound quantitatively killed larvae at the low micromolar levels (Table 1). Moreover, to explore the optimal hydrophobicity of the phenanthroline structure for enhancing membrane penetration, we examined the IC_50_ and IC_90_ values of PHN-Me, PHN-*p*-Me_2_, and PHN-Ph_2_ (Fig. 1 and Table 1), whose protons at the 4-, 5-, or 7-positions were individually substituted with methyl or phenyl groups. Comparing the IC_50_ values revealed that the larvicidal activity of PHN-*p*-Me_2_ with two methyl groups was more than three times higher than that of PHN with no substitutional groups. Meanwhile, PHN-Ph_2_ with two phenyl groups, whose hydrophobicity was stronger than that of the methyl groups in PHN-*p*-Me_2_, showed no larvicidal activity. Previously, PHN-*p*-Me_2_ and PHN-Ph_2_ led to the loss and enhancement of antiamebic activity against *E. histolytica*, respectively [17]. This contrasting phenomenon suggests that the difference in hydrophobic and steric properties, which are influenced by substitutional groups, is an important factor in determining the specificity in the effects of PHN-*X* against pathogenic parasites.

**Table 1:** In vitro larvicidal activities of DFO and PHN-*X* against the schistosomula of *S. mansoni*

Compound	IC_50_ (μM)	IC_90_ (μM)
DFO	> 50	> 50
PHN	6.90 ± 0.06	9.28 ± 0.01
PHN-Me	6.35 ± 0.49	9.13 ± 0.15
PHN-*p*-Me_2_	2.05 ± 0.06	4.18 ± 0.02
PHN-Ph_2_	> 50	> 50
PHN-*o*-Me_2_	18.5 ± 0.3	41.0 ± 0.1
PHN-(OMe)_2_	2.10 ± 0.12	4.27 ± 0.15
PHN-(NH_2_)_2_	1.51 ± 0.22	3.93 ± 0.12
PHN-(NMe_2_)_2_	1.98 ± 0.05	4.16 ± 0.02

The IC_50_ and IC_90_ values are shown as the means with standard deviation obtained from three independent experiments

Next, the IC_50_ and IC_90_ values of PHN-*o*-Me_2_ (Fig. 1) were assessed to confirm whether the iron-binding ability of PHN-*p*-Me_2_ contributes to its larvicidal activity. The molecular formula and hydrophobicity of PHN-*o*-Me_2_ are the same as those of PHN-*p*-Me_2_; however, the two methyl groups are located at the *ortho* positions of the pyridyl nitrogen atoms. Hence, when more than one PHN-*o*-Me_2_ approach an Fe(II) ion to form an iron complex, steric hindrance between the intermolecular methyl groups likely occurs around the iron ion. This repulsion can increase the length of the coordination bonds between two nitrogen atoms of PHN-*o*-Me_2_ and the iron center, leading to instability of the resulting ferrous complex. As expected, the IC_50_ and IC_90_ values of PHN-*o*-Me_2_ were much higher than those of PHN-*p*-Me_2_, indicating a decrease in the larvicidal activity (Table 1). This observation aligns with the hypothesis that the larvicidal effect of PHN-*p*-Me_2_ is associated with its iron-binding ability. In a recent study, we uncovered that the antiplasmodial activity of terpyridine-based compounds is influenced by the electronic properties of their iron-binding pyridyl nitrogen atoms, which are determined by substitutional groups located at their *para* positions [22]. Consequently, we examined PHN-(OMe)_2_, PHN-(NH_2_)_2_, and PHN-(NMe_2_)_2_, which have electron-donating methoxy, amino, and dimethyl amino groups at the *para* positions of their two nitrogen atoms, respectively, to enhance the larvicidal activity of PHN-*X* through an electronic substituent effect (Fig. 1). No significant differences in the IC_50_ and IC_90_ values were observed among the three compounds (Table 1); however, PHN-(NH_2_)_2_ showed a slightly higher larvicidal activity than that of PHN-*p*-Me_2_. Nonetheless, when comparing the IC_90_ values, the four compounds, including PHN-*p*-Me_2_, caused significant larval death, surpassing the effectiveness of PZQ (IC_90_ = 110.4 μM) by over 25 times [23].

### PHN-X inhibits egg production by paired adult worms of *S. mansoni* in vitro

**Fig. 2:**
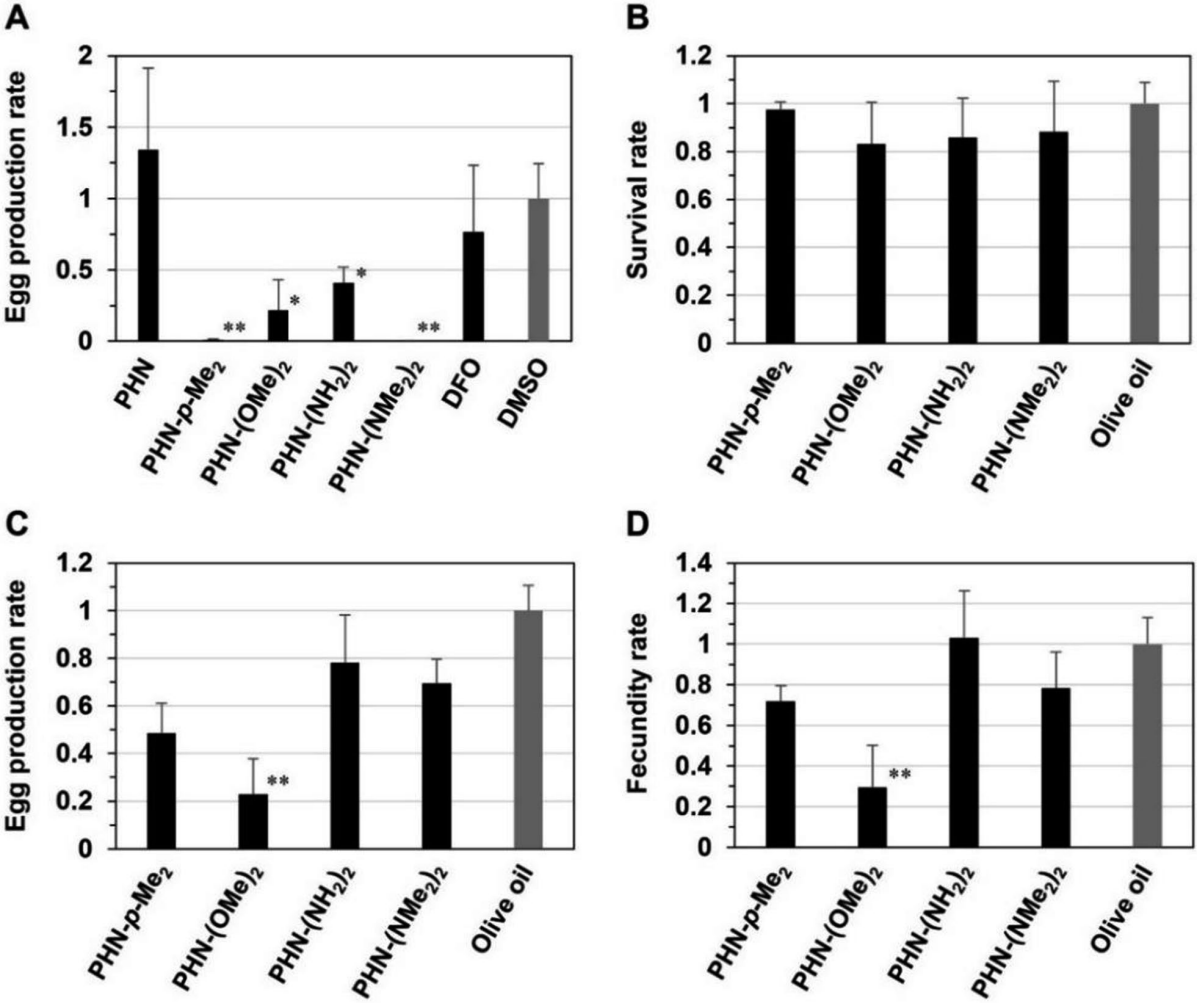
**A** Production rate of eggs laid by paired adult worms of *S. mansoni* after in vitro treatment with PHN, PHN-*X*, DFO, or DMSO for 72 h. The rates were calculated based on the number of eggs upon treatment with DMSO alone as a control. **B** Survival rate of adult worms in the portal vein of mice at 6-week post-infection after the oral administration of PHN-*X*. **C** Production rate of eggs and **D** fecundity rate of female adult worms in the liver and intestine of mice at 6-week post-infection treated in the same manner. Fecundity was determined by dividing the number of eggs with the number of female adult worms. The rates were calculated based on mice at 6-week post-infection after the oral administration of olive oil alone as a control. The data are shown as the means with standard deviation obtained from three independent experiments. *p < 0.05, **p < 0.001 (Student’s *t* test)

Based on the general protocol [21], we investigated the effects of PHN-*X* with high larvicidal activity, PHN-*p*-Me_2_, PHN-(OMe)_2_, PHN-(NH_2_)_2_, and PHN-(NMe_2_)_2_, on adult worms of *S. mansoni* and the egg production in vitro. At the concentration of 5 μM, none of the tested compounds inhibited the survival of the adult worms. Subsequently, after adding red blood cells to the culture media to supply nutrients and iron ions, which promote the sexual maturation of the adult worms and induce egg-laying [9, 10], egg production rates were calculated by counting the eggs excreted after treatment with or without each compound. As shown in Fig. 2a, neither DFO nor PHN suppressed egg production; nevertheless, the compounds partially affected the egg-laying process of the schistosomes at higher concentrations [16, 24]. Probably, the two compounds may be susceptible to red blood cells, which were used to replicate in vivo growing environments of adult worms. Meanwhile, all four PHN-*X* exhibited strong inhibitory effects on egg production (Fig. 2a). Notably, the rate after treatment with PHN-*p*-Me_2_ or PHN-(NMe_2_)_2_ was less than one-hundredth of that observed in the control or PHN. Thus, the tested PHN-*X* could block the survival of schistosomula and egg production of paired adult worms in vitro.

### PHN-(OMe)_2_ exhibits anti-fecundity effect against female adult worms of *S. mansoni* in vivo

To determine the effects of PHN-*p*-Me_2_, PHN-(OMe)_2_, PHN-(NH_2_)_2_, and PHN-(NMe_2_)_2_ on the survival capacity of adult worms in vivo, these compounds were orally administered to mice infected with *S. mansoni* cercariae. After administration of PHN-*X* into the infected mice, no serious side effects such as death, spasm, or behavior disorders were observed. Furthermore, the dosages of PHN-*X* used in the experiments were estimated to be within a range that minimizes the potential risk of anemia. As shown in Fig. 2b, regardless of the type of compound, no change was observed in the number of adult worms recovered from the portal vein of the infected mice. Moreover, in contrast to the observation that the all four PHN-*X* suppressed egg production in vitro (Fig. 2a), only PHN-(OMe)_2_ significantly reduced the number of eggs in the liver and intestine (Fig. 2c). This reduction led to a significant decline in the fecundity rate of female adult worms (Fig. 2d). Under neutral aqueous conditions, PHN-(NH_2_)_2_ and PHN-(NMe_2_)_2_ are positively charged through protonation of the amine groups with strong basicity. The positive charges lead to a tendency to interact with the negative charges of cell membranes in vivo. The electrostatic interaction may prevent the two compounds from reaching the adult worms, resulting in a drastic decrease in the anti-fecundity effect. Meanwhile, PHN-*p*-Me_2_ possesses hydrophobic and non-polar methyl groups, which cause it to adsorb onto serum albumins abundant in blood. In general, the hydrophobic adsorption reduces the concentration of its free form, limiting its potential effectiveness. Thus, PHN-(OMe)_2_, which has unprotonated and polar methoxy groups, was the most suitable compound for expressing anti-fecundity effects in vivo when administered orally to infected mice.

**Fig. 3:**
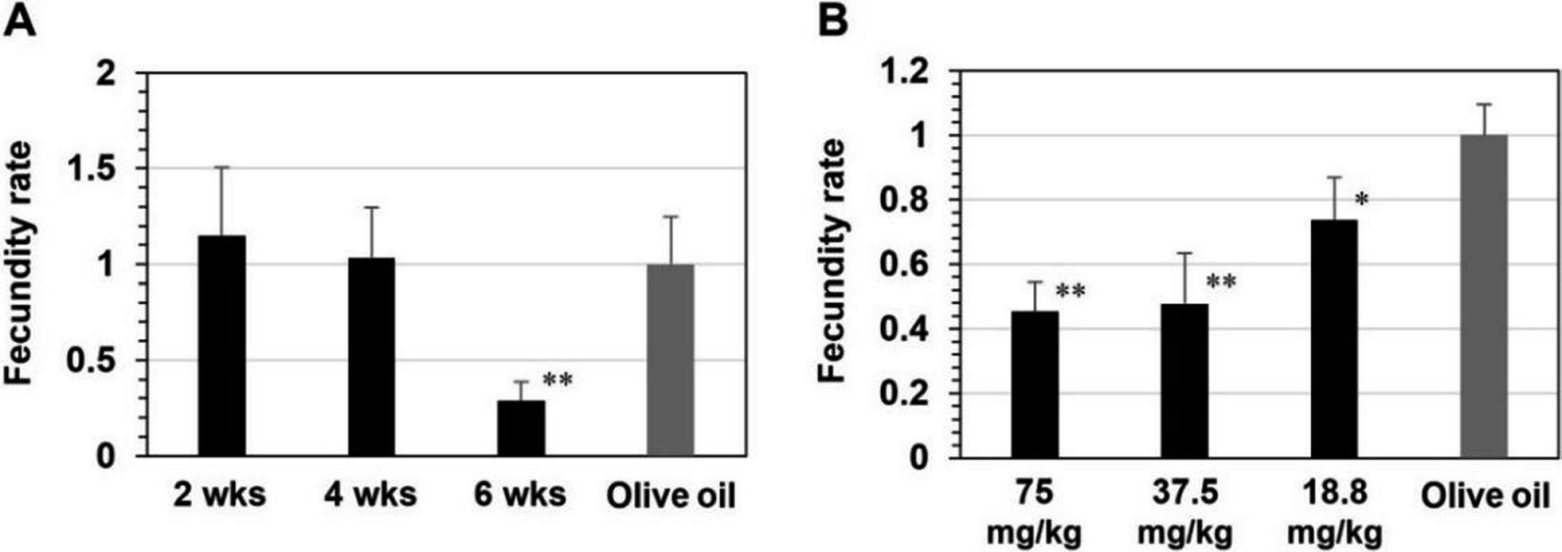
**A** Stage- and **B** dose-dependence on the fecundity rate of female adult worms in *S. mansoni*-infected mice after the oral administration of PHN-(OMe)_2_. Adult worms and eggs were collected from the liver and intestine of each mouse at 8 weeks after infection. Fecundity was determined by dividing the number of eggs with the number of female worms. The rates were calculated based on post-infection mice after the oral administration of olive oil alone as a control and are shown as the means with standard deviation obtained from three independent experiments. *p < 0.05, **p < 0.001 (Student’s *t* test)

Furthermore, to identify the stage of adult worms at which PHN-(OMe)_2_ exerts anti-fecundity effects in vivo, the compound was orally administered to mice that had adult worms at the pre-liver (2 weeks after infection), pre-egg-laying (4 weeks after infection), or egg-laying (6 weeks after infection) stages. Subsequently, adult worms and eggs were collected from the mouse livers and intestines, and the numbers were counted at each stage. PHN-(OMe)_2_ did not affect the survival rate of adult worms at any stage (Fig. S1a) but decreased the egg production rate at the egg-laying stage (Fig. S1b). As a reflection of these phenomena, the fecundity rate of female adult worms was significantly reduced at the same stage (Fig. 3a). Furthermore, the anti-fecundity activity of PHN-(OMe)_2_ was dependent on the dose administered to the infected mice (Fig. 3b). Overall, these data indicate that PHN-(OMe)_2_ influences the function of the reproductive organs in adult worms in vivo, without affecting their growth.

### PHN-(OMe)_2_ atrophies ovaries of female adult worms by containing Fe(II) ions

**Fig. 4:**
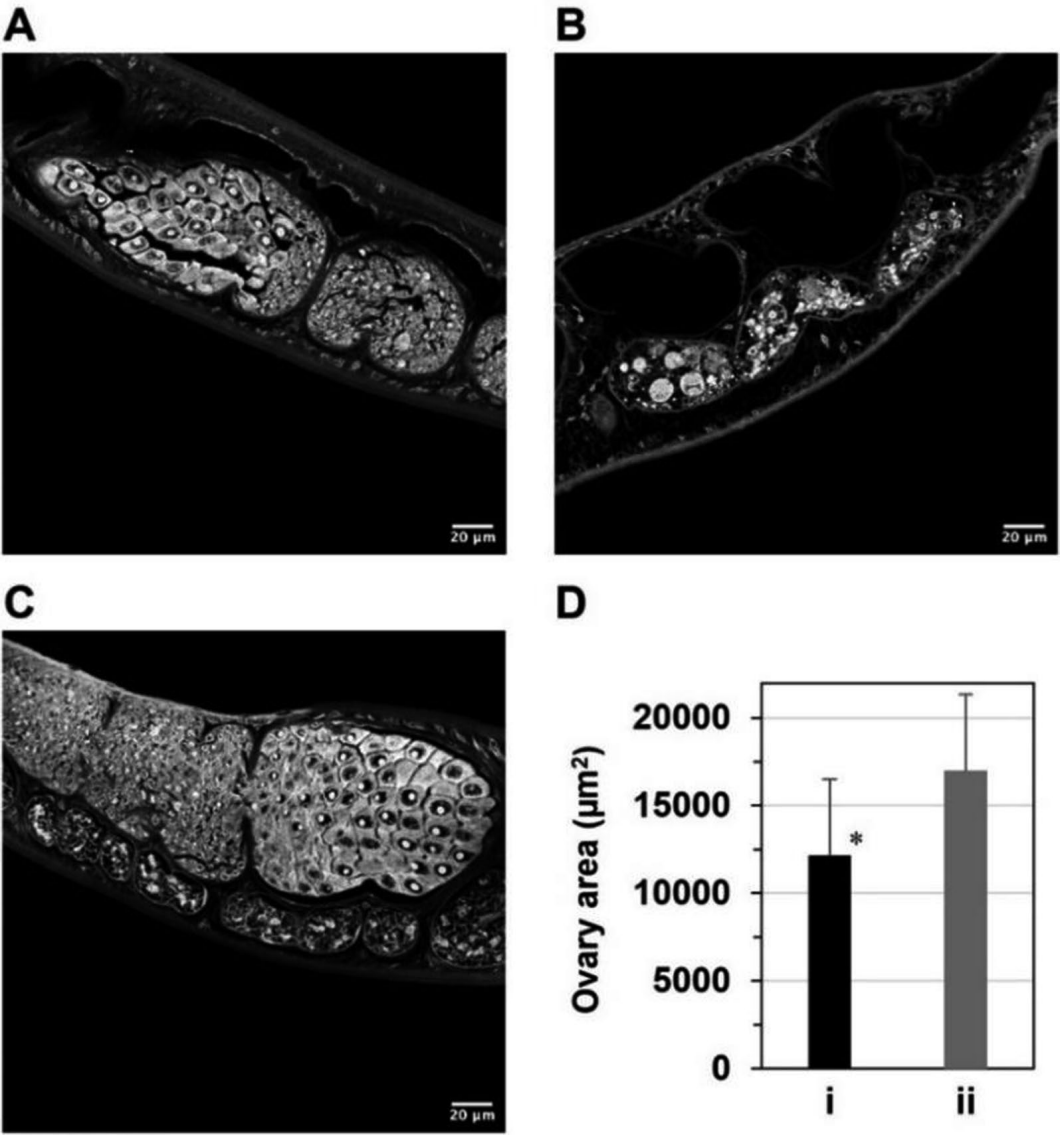
Images of the ovaries of female adult worms collected from *S. mansoni*-infected mice after the oral administration of (**A** and **B**) PHN-(OMe)_2_ or (**C**) olive oil as a control. (**D**) Comparison of the ovarian area (mm^2^) of female adult worms in infected mice treated with (i) PHN-(OMe)_2_ or (ii) olive oil. The data are shown as the means with standard deviation obtained from ten randomly selected female worms. *p < 0.005 (Student’s *t* test)

**Fig. 5:**
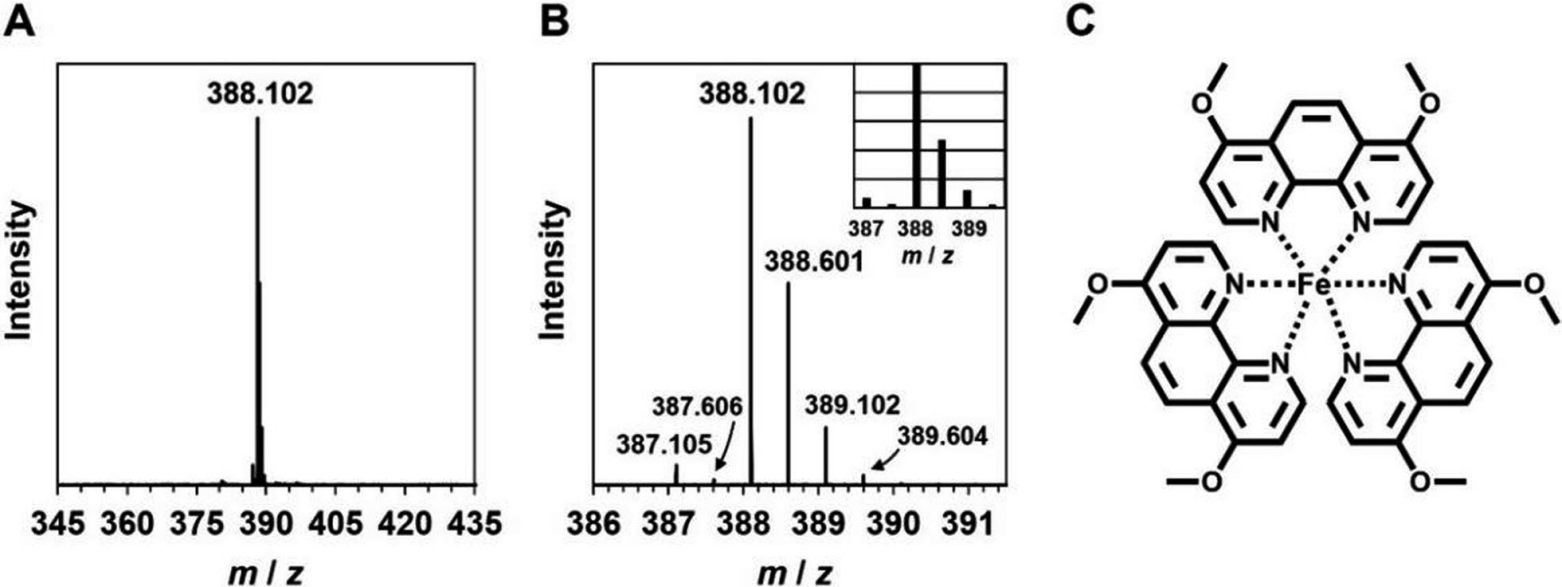
ESI–MS spectroscopic analysis of the iron complex formed through the reaction of PHN-(OMe)_2_ with Fe(II) ions. **A,** **B** Positive ion peak clusters and isotope pattern observed at *m/z* 388.102 corresponded to (B: inset) the calculated those of 1:3 Fe(II):PHN-(OMe)_2_ complex formulated as **C** [Fe(PHN-(OMe)_2_)_3_]^2+^, respectively

To investigate whether PHN-(OMe)_2_ impacts the ovaries of female adult worms, we monitored their morphological changes in *S. mansoni*-infected mice after administering the compound orally or olive oil as a control. Microscopic observations revealed that the ovaries were significantly atrophied (Fig. 4a, b) compared to the control one (Fig. 4c). The reduction in the ovarian area (Fig. 4d) proves that this ovarian disruption was directly responsible for impairing egg production of female adult worms. In addition, electrospray ionization mass (ESI–MS) spectroscopic analysis showed that PHN-(OMe)_2_ rapidly bound to an Fe(II) ion in solution, forming a stable ferrous complex formulated as [Fe(PHN-(OMe)_2_)_3_]^2+^ even under an O_2_ atmosphere (Fig. 5). Thus, these data reveal that PHN-(OMe)_2_ can induce abnormal ovarian atrophy in vivo by containing Fe(II) ions, which are essential for the maturation of prelaying female adult worms.

## Conclusion

In this study, to explore the potential drug effects of phenanthroline-based compounds against *S. mansoni*, which has a diverse life cycle, we first verified whether DFO and PHN-*X* could kill schistosomula in vitro. Based on their IC_50_ and IC_90_ values, while DFO favorably binds to Fe(III) ions through negatively charged oxygen atoms, the natural siderophore did not affect the survival of larvae. Conversely, PHN-*X*, which specifically binds to Fe(II) ions through neutral nitrogen atoms, was effective in causing larval death. While comparing the physicochemical properties of DFO and PHN-*X*, we found that the electrochemically neutral and low-molecular-weight compounds with iron-binding affinity have the advantage of penetrating larval membranes and subsequently containing intracellular iron ions. Notably, the larvicidal activity of PHN-*X* depended on the strength of the coordination bonds between its two nitrogen atoms and an iron ion, and tended to be enhanced by introducing electron-donating groups substituted at the 4- and 7-positions of the phenanthroline structure. Furthermore, PHN-*p*-Me_2_, PHN-(OMe)_2_, PHN-(NH_2_)_2_, and PHN-(NMe_2_)_2_ strongly inhibited the egg-laying behavior of paired adult worms in vitro. In oral administration of the four PHN-*X* into *S. mansoni*-infected mice under various conditions, only PHN-(OMe)_2_ successfully decreased the egg production rate in the liver and intestine, resulting in a significant reduction in the fecundity rate of female adult worms at the egg-laying stage. Finally, morphological observations of the reproductive organs extracted from female adult worms revealed that PHN-(OMe)_2_ specifically caused abnormal ovarian atrophy, which contributed to the inhibition of egg production in vivo. Thus, the ability of PHN-(OMe)_2_ to capture iron ions significantly impacted the survival capacity of schistosomula and the egg-laying organs of female adult worms. Based on these findings, the strategy for containing the iron source essential for *S. mansoni* could offer insight for developing next-generation drugs aimed at diversifying the treatment of schistosomiasis or suppressing the transmission of *Schistosoma* infections.

## Supplementary Information

Additional file 1.

## Data Availability

No data sets were generated or analyzed during the current study.

## Abbreviations

ART: Artemisinin derivatives
DFO: Deferoxamine
PHN: Phenanthroline
PZQ: Praziquantel
WHO: World Health Organization
