# Supplementary material for: Molecular containment of iron source inhibits larval survival of Schistosoma mansoni and egg-laying behavior of the female adult worms via ovarian atrophy
Source: Trop Med Health. 2025 Sep 2;53:121. doi: 10.1186/s41182-025-00800-x (PMC12403254; doi:10.1186/s41182-025-00800-x)
Supplement: Supplementary file 1 — Additional file 1. [file 41182_2025_800_MOESM1_ESM.pdf]

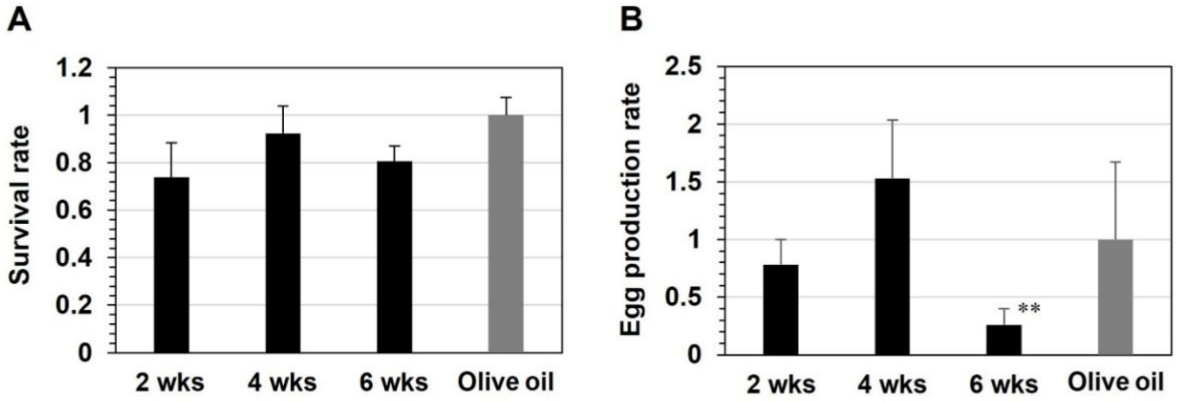

Fig. S1 Stage dependence on (A) the survival rate of adult worms of *S. mansoni* and (B) production rate of eggs laid by paired adult worms in post-infection mice after the oral administration of PHN-$(OMe)_2$. The compound was individually administered to mice with adult worms at the pre-liver (two weeks after infection), pre-egg-laying (four weeks after infection), or egg-laying (six weeks after infection) stages. Adult worms and eggs were collected from the liver and intestine of each mouse at eight weeks after infection. The rates were calculated based on post-infection mice after the oral administration of olive oil alone as a control and are shown as the means with standard deviation obtained from three independent experiments. $^{**}p < 0.001$ (Student's t-test).
